# Astrocytic JWA deletion exacerbates dopaminergic neurodegeneration by decreasing glutamate transporters in mice

**DOI:** 10.1038/s41419-018-0381-8

**Published:** 2018-03-02

**Authors:** Rihua Wang, Xue Zhao, Jin Xu, Yifan Wen, Aiping Li, Ming Lu, Jianwei Zhou

**Affiliations:** 1 Departemnt of Molecular Cell Biology & Toxicology, School of Public Health, Nanjing Medical University, 101 Lonamian Avenue, Jiangning District, Nanjing, 211166 China; 2 Neuroprotective Drug Discovery Key Laboratory, Department of Pharmacology, Nanjing Medical University, 101 Longmian Avenue, Jiangning District, Nanjing, 211166 China

## Abstract

Astrocytic JWA exerts neuroprotective roles by alleviating oxidative stress and inhibiting inflammation. However, the molecular mechanisms of how astrocytic JWA is involved in dopaminergic neurodegeneration in Parkinson’s disease (PD) remain largely unknown. In this study, we found that astrocyte-specific JWA knockout mice (JWA CKO) exacerbated dopamine (DA) neuronal loss and motor dysfunction, and reduced the levels of DA and its metabolites in a 1-methyl-4-phenyl-1, 2, 3, 6-tetrahydropyridine/probenecid (MPTP/p)-induced PD model. Astrocytic JWA deficiency repressed expression of excitatory amino-acid transporter 2 (GLT-1) and glutamate uptake both in vivo and in vitro. Further, the regulation of GLT-1 expression was involved in JWA-triggered activation of the MAPK and PI3K signaling pathways. JWA-increased GLT-1 expression was abolished by inhibitors of MEK and PI3K. Silencing CREB also abrogated JWA-increased GLT-1 expression and glutamate uptake. Additionally, JWA deficiency activated glial fibrillary acidic protein (GFAP), and increased the expression of STAT3. Similarly to the MPTP model, paraquat (PQ) exposure produced PD-like phenotypes in JWA CKO mice. Taken together, our findings provide novel insights into astrocytic JWA function in the pathogenesis of neurotoxin mouse models of PD.

## Introduction

Parkinson’s disease (PD) is one of the most common and progressive neurodegenerative movement disorders. PD is characterized by the selective death of dopamine (DA)-containing neurons in the substantia nigra compact (SNc)^1^. Dopaminergic neuron loss leads to motor abnormalities, including bradykinesia, rigidity, rest tremor, gait, and postural instability^2,3^. Although it is widely known that oxidative stress, excitotoxicity, inflammation, apoptosis, and mitochondrial dysfunction play important roles in PD, the exact mechanism of dopaminergic neuron death remains to be further elucidated^4,5^.

In the mammalian central nervous system (CNS), astrocytes are the most abundant glial cells with many critical physiological functions^6^. During progressive disease and acute injuries, astrocytes become reactive in responses to all pathological conditions^7^. In a 1-methyl-4-phenyl-1, 2, 3, 6-tetrahydropyridine (MPTP) model and post-mortem Parkinsonian brains, astrocyte reactivity parallels the time course of dopaminergic neuron loss in the SNc^8^. Glutamate is the major excitatory transmitter in the CNS. The clearing of extracellular glutamate and maintaining glutamate homeostasis through membrane excitatory amino-acid transporters (EAATs) are important functions of astrocytes. Impaired glutamate uptake by astrocytes can result in neuron cell death due to excessive levels of glutamate and excitatory toxicity, and is implicated in several neurodegenerative diseases, including Alzheimer’s disease (AD) and PD^9,10^. EAAT1/GLAST and EAAT2/GLT-1 are highly specific glutamate transporters that are expressed on astrocytes. Under physiological conditions, extracellular excitatory neurotransmitter clearance is primarily performed by GLT-1. Reduced GLT-1 expression contributes to the comorbidity of depression and anxiety^10^ and aggravates effects of traumatic injuries^11^. The dysregulation of GLT-1 is associated with neuronal damage in neurodegenerative diseases^12^.

JWA, also known as ADP-ribosylation-like factor 6 interacting protein 5 (ARL6ip5), is a multifunctional cytoskeleton-binding protein, induced by all-trans retinoic acid^13^. JWA gene homologs in rat and murine are the glutamate transporter-associated protein 3–18 (GTRAP3-18) and addicsin^14,15^. Addicsin is ubiquitously expressed in numerous tissues, and is expressed at a higher level in the CNS^16^. Previous studies have demonstrated that GTRAP3-18 can specifically interact with EAAT3-mediated glutathione synthesis, but not with the other types of glutamate transporters^17^. Recent studies have revealed that JWA deficiency through the phosphatidylinositol 3-kinase (PI3K)/protein kinase B (Akt)/mammalian target of rapamycin (mTOR) pathway increases newborn neurons and enhances spatial cognitive potentiation in mice^18^. Gain- and loss-of-function studies revealed that astrocytic JWA exerts neuroprotective roles via the alleviation of oxidative stress and the inhibition of inflammation^19^. Taken together, all available evidence suggests that JWA has critical roles in physiological and pathological processes in the CNS. However, if JWA plays a role in PD models remain to be investigated.

In this study, we used the Cre-loxP system under regulation of the mouse glial fibrillary acidic protein (GFAP) promoter to conditionally delete JWA from astrocytes. We then established PD models to investigate the influence of JWA deficiency on dopaminergic neuron survival. We further explored the molecular mechanisms of JWA in neurotoxin-induced neurodegeneration, with a focus on the regulation of glutamate transporters.

## Results

### Selective JWA deletion in astrocytes exacerbates motor dysfunction in the MPTP/probenecid mouse model

**Fig. 1:**
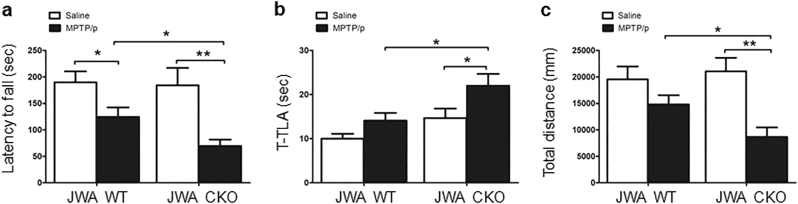
Astrocytic JWA deficiency exacerbates MPTP/p-induced motor deficits. **a** JWA WT and JWA CKO mice were trained 3 days on the rotarod until their motor performance was assessed. The time spent on the rod was calculated (in seconds). **b** Behavioral assays of climbing time in pole test, the time taken for mice to descend a pole (T-TLA). **c** The moved distance in an open field within 10 min in JWA WT and JWA CKO mice were recorded by the Top View Animal Behavior Analyzing System. *n* = 15. Data are presented as mean ± SEM, one-way ANOVA, *P < 0.05, **P < 0.01

Astrocyte-specific JWA deletion mice (JWA CKO) were generated as described previously^19^ (Supplementary Figure 1a-c). The expression levels of JWA were very low in the JWA CKO mice brain tissues compared with that in wild-type (JWA WT) mice (Supplementary Figure 1d). To determine whether JWA deletion in astrocytes exacerbated motor dysfunction in the MPTP/probenecid (MPTP/p) mouse model, mice was evaluated by monitoring locomotion ability. In the rotarod test, MPTP/p treatment significantly decreased latency on the rotarod in both JWA WT (*P* < 0.05) and JWA CKO mice (*P* < 0.01). Compared with JWA WT mice, MPTP/p treatment induced a 44% greater decrease of the latency time on the rod in JWA CKO mice (*P* < 0.05; Fig. 1a). In the pole test, MPTP/p treatment significantly increased the total time spent on the pole in JWA CKO mice compared with untreated JWA CKO mice (*P < *0.05). In comparison with MPTP/p-treated JWA WT mice, there was a 1.56-fold (*P* < 0.05) increase in response to MPTP/p treatment in JWA CKO mice (Fig. 1b). In the open-field test, after chronic MPTP/p administration, JWA CKO mice traveled only ~ 42% of the distance traveled by the JWA WT mice (*P* < 0.05; Fig. 1c). Overall, astrocytic JWA deficiency exacerbated the motor dysfunction induced by chronic MPTP/p exposure.

### Deletion of astrocytic JWA aggravates dopaminergic neurodegeneration in the MPTP/p mouse model

**Fig. 2:**
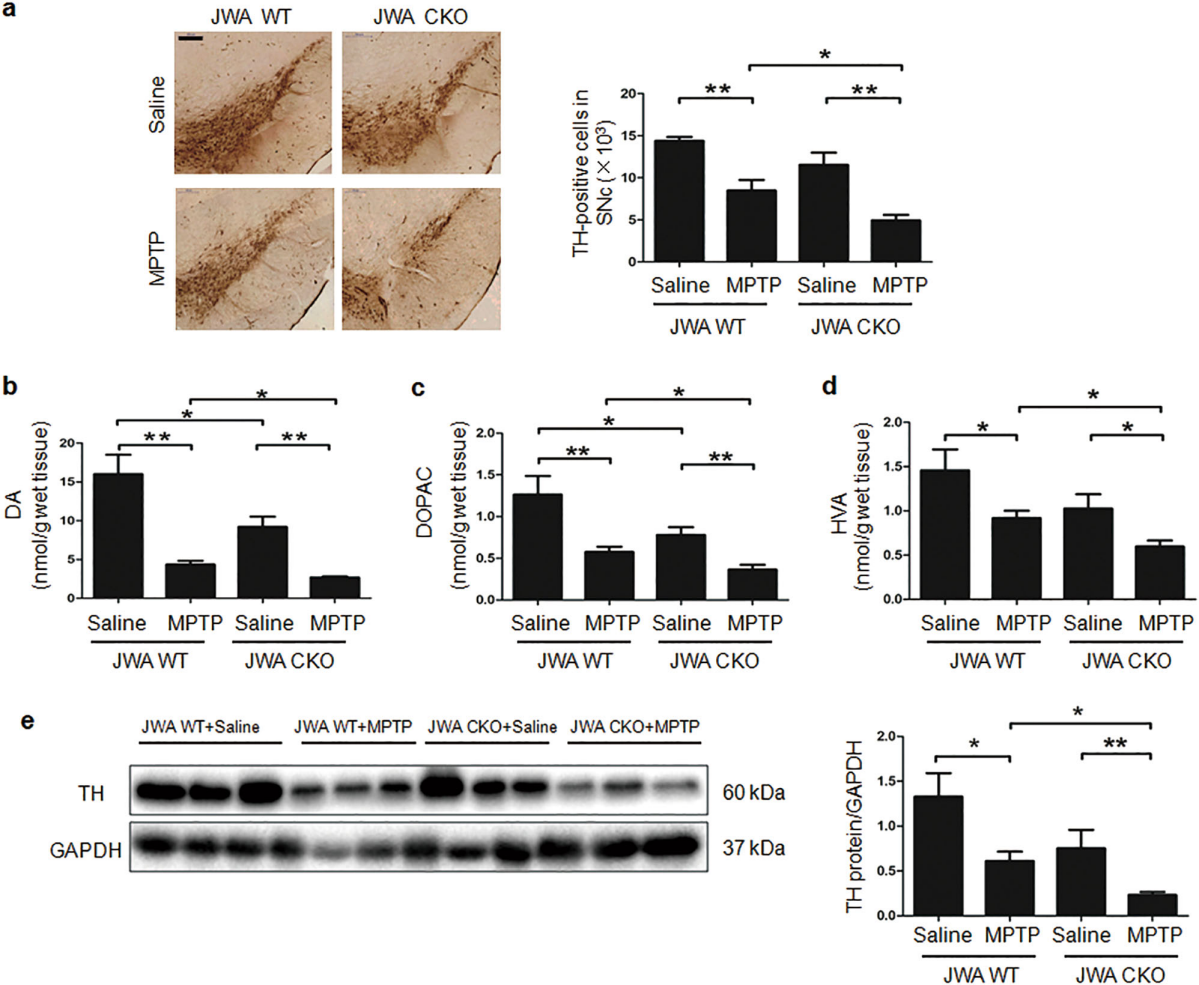
Astrocytic JWA deletion decreases the number of TH-positive neurons and exacerbates MPTP/p-neurotoxicity in mice. **a** Immunohistochemical staining and quantitative data for TH-positive neurons in the SNc (*n* = 4). Scale bar = 200 µm. **b-d** Levels of DA, DOPAC, and HVA were analyzed by HPLC coupled to electrochemical detection in striatum tissues (*n* = 8). **e** Western blotting and quantification for analysis of TH protein expression in midbrain extracts. GAPDH was used as a loading control to confirm that equal amounts of protein were loaded in each line (*n* = 7). Data are presented as mean ± SEM, one-way ANOVA, *P < 0.05, **P < 0.01

To further confirm the MPTP/p-induced dopaminergic neuron death of astrocytic JWA deletion mice, tyrosine hydroxylase (TH) immunohistochemical staining in SNc was performed (Fig. 2a). The stereological counts showed that MPTP/p treatment decreased the number of TH-positive cells by 41% in JWA WT mice, but by 57% in JWA CKO mice (*P* < 0.01). Compared with the MPTP/p-treated JWA WT mice, the number of TH-positive cells were reduced ~ 42% in MPTP/p -treated JWA CKO mice (*P* < 0.05).

High performance liquid chromatography (HPLC) analysis of the striatum (STR) tissues was performed to measure monoamine neurotransmitters including DA and its metabolites. In agreement with the immunohistochemical results, JWA CKO mice had a lower basal expression of TH, which was reflected by reduced basal levels of striatal DA and dihydroxyphenylacetic acid (DOPAC) content (*P* < 0.05; Figs. 2b, c). After MPTP/p treatment, the levels of DA, DOPAC, and homovanillic acid (HVA) were lower in the JWA CKO mice than in JWA WT mice (*P* < 0.05; Figs. 2b-d). Moreover, immunoblots confirmed that JWA CKO mice had a lower TH in the STR than did the JWA WT mice after chronic MPTP/p administration (*P* < 0.05; Fig. 2e).

### Reduced expression of GLT-1 in astrocytic JWA-deficient mice

**Fig. 3:**
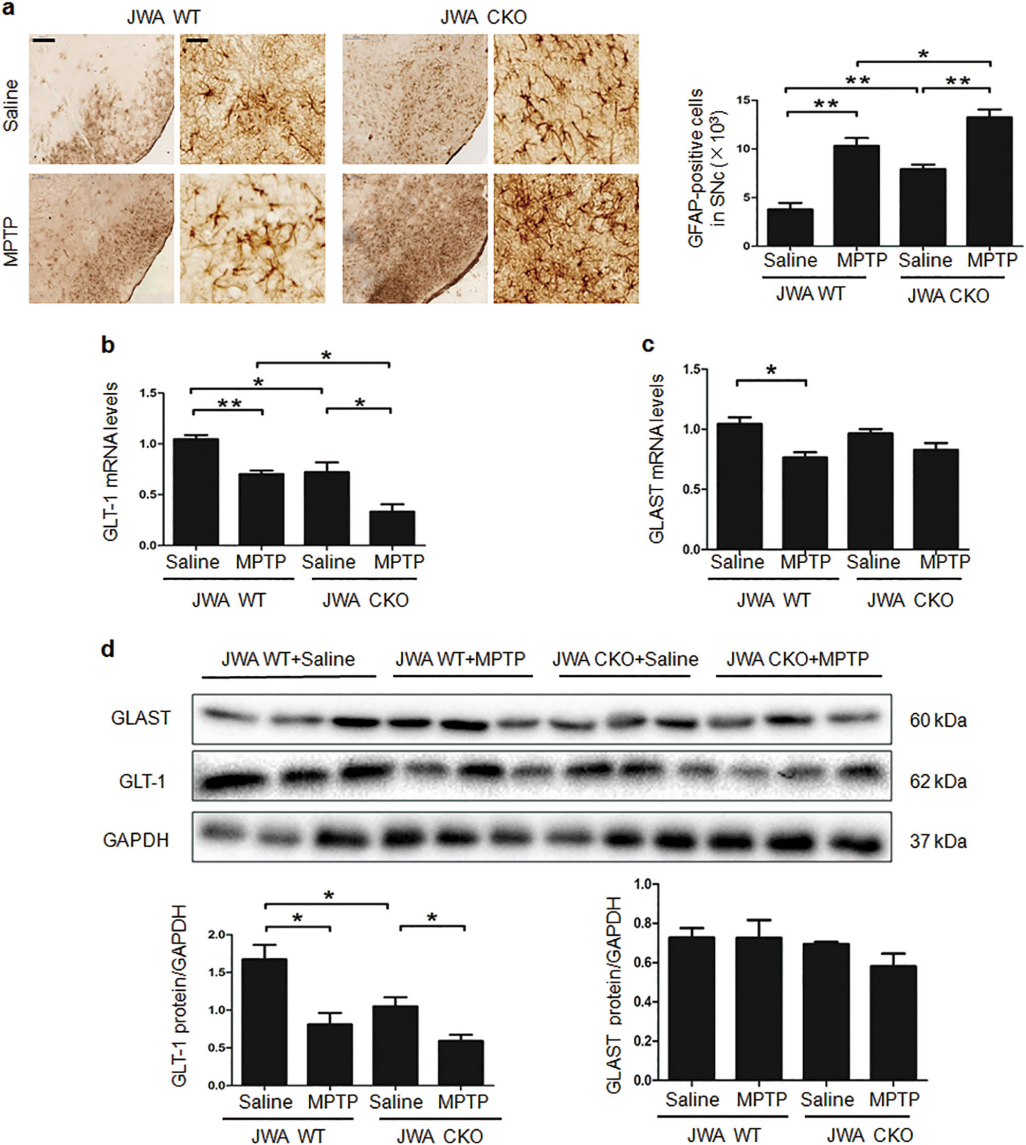
Astrocytic JWA deficiency induces astrocyte activation and reduced the expression of GLT-1 in vivo. **a** Immunohistochemical staining and quantitative data for GFAP-positive neurons in the SNc (*n* = 4). Scale bars = 200 μm (low magnification) and 50 μm (high magnification). **b**, **c** Quantitative real-time PCR analyses for GLT-1 and GLAST are shown in the SNc. GAPDH was used as a control to normalize the differences in the amount of total RNA in each sample (*n* = 6). **d** Western blotting and quantification for analysis of GLT-1 and GLAST protein expression in midbrain extracts. GAPDH was used as a loading control to confirm that equal amounts of protein were loaded in each line (*n* = 7). Data are presented as mean ± SEM, one-way ANOVA, *P < 0.05, **P < 0.01

Deletion of astrocytic JWA showed an obvious 2.1-fold increase in the number of GFAP-positive cells compared with the JWA WT mice (*P* < 0.01). In comparison with the MPTP/p-treated JWA WT mice, the number of GFAP-positive cells was increased 1.28-fold in MPTP/p -treated JWA CKO mice (*P* < 0.05; Fig. 3a).

To examine a possible correlation between astrocytic JWA expression and EAATs in the MPTP/p mouse model, we used quantitative real-time PCR (qPCR) assay to measure GLAST and GLT-1 mRNA expression in SNc. The GLT-1 in JWA CKO mice was decreased by 32% in comparison with JWA WT mice without MPTP/p treatment (*P* < 0.05). In comparison with MPTP/p-treated JWA WT mice, GLT-1 was decreased by 53% in MPTP/p-treated JWA CKO mice (*P* < 0.05). No significant difference was found in GLAST between JWA WT mice and JWA CKO mice (Figs. 3b, c). At the protein level, even in the absence of MPTP/p, JWA CKO mice had a lower basal expression of GLT-1 than untreated JWA WT mice (*P* < 0.05). MPTP/p treatment decreased GLT-1 expression by 33% compared with JWA WT mice, vs. 54% for JWA CKO mice (*P* < 0.05). However, there was no difference in GLAST for the JWA CKO mice and JWA WT mice (Fig. 3d). These results indicated that astrocytic JWA deficiency decreased the production of GLT-1 at both the transcript and protein levels.

### JWA regulates GLT-1 in primary culture astrocytes

**Fig. 4:**
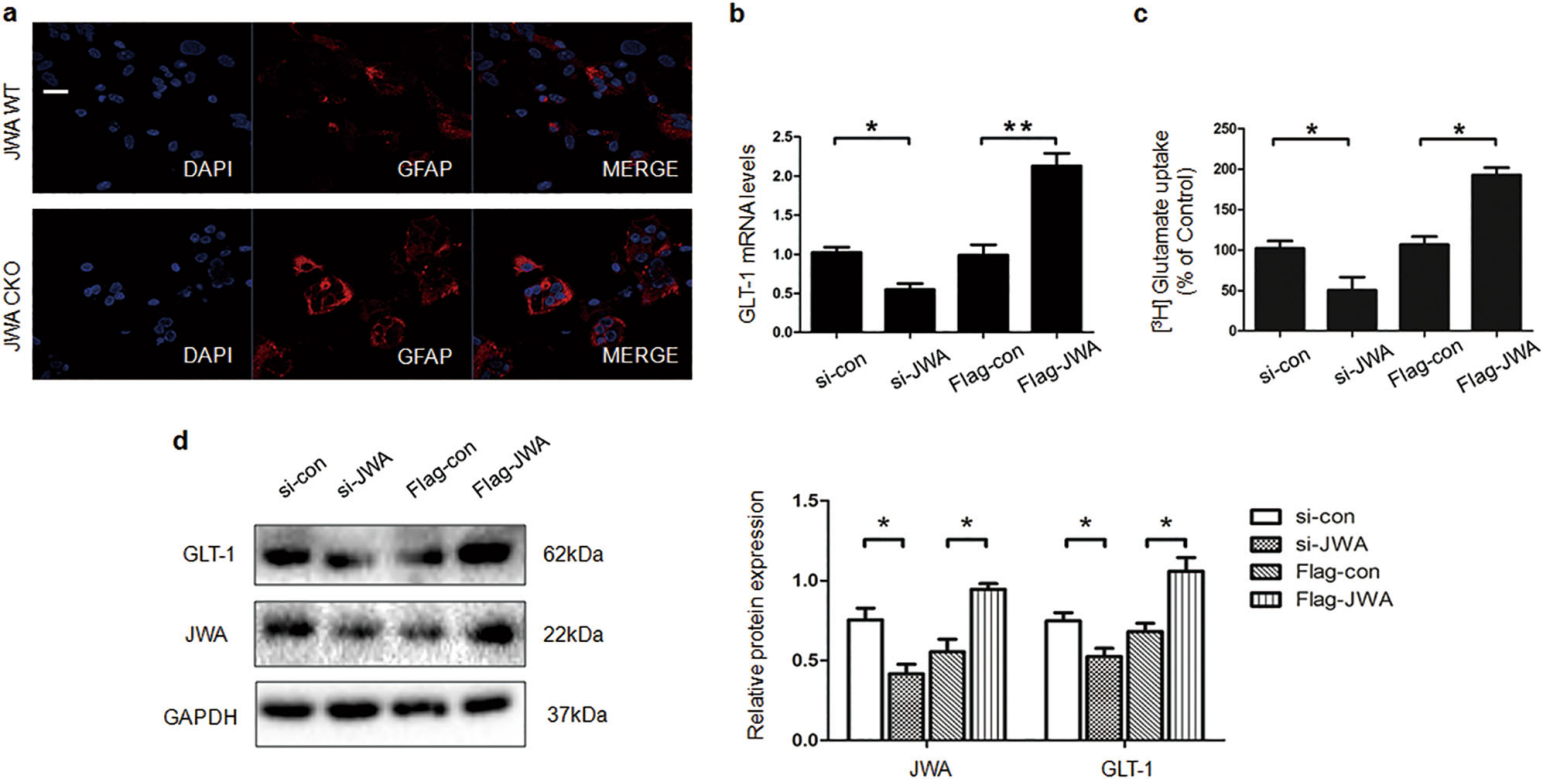
Effect of JWA on astrocyte reactivity and GLT-1 expression in primary cultured astrocytes. **a** After primary cultured astrocytes were isolated from JWA WT and JWA CKO, immunofluorescence for GFAP (red) was immunostained with anti-GFAP antibody. Scale bars = 20 μm. **b** The primary cultured astrocytes were transfected with si-JWA or Flag-JWA. After 48 h, the mRNA levels of GLT-1 were measured by quantitative real-time PCR. GAPDH was used as a control to normalize the differences in the amount of total RNA in each sample (*n* = 6). **c** [^3^H]-Glutamate uptake assay was performed after primary astrocytes were transfected with si-JWA or Flag-JWA (*n* = 3). **d** Representative western blotting results were detected using anti-GLT-1 antibody. GAPDH was used as a loading control to confirm that equal amounts of protein were loaded in each line (*n* = 3). Data are presented as mean ± SEM, one-way ANOVA, *P < 0.05, **P < 0.01

To further confirm effects of JWA on primary astrocytes, GFAP immunofluorescence staining was performed. GFAP was significantly elevated in primary astrocytes of JWA CKO mice compared with JWA WT mice (Fig. 4a). Our results also showed that knocked down or overexpressed JWA significantly downregulated or elevated, respectively, GLT-1 protein and mRNA expression levels compared with the levels in the vector control in primary astrocytes (*P* < 0.05 and *P* < 0.01, respectively; Figs. 4b, d). Moreover, JWA overexpression led to a 1.81-fold increase in glutamate uptake activity (*P* < 0.05), but JWA knock down led to a decrease of 50% (*P* < 0.05; Fig. 4c).

### JWA regulates GLT-1 expression via multiple signaling pathways in vitro

**Fig. 5:**
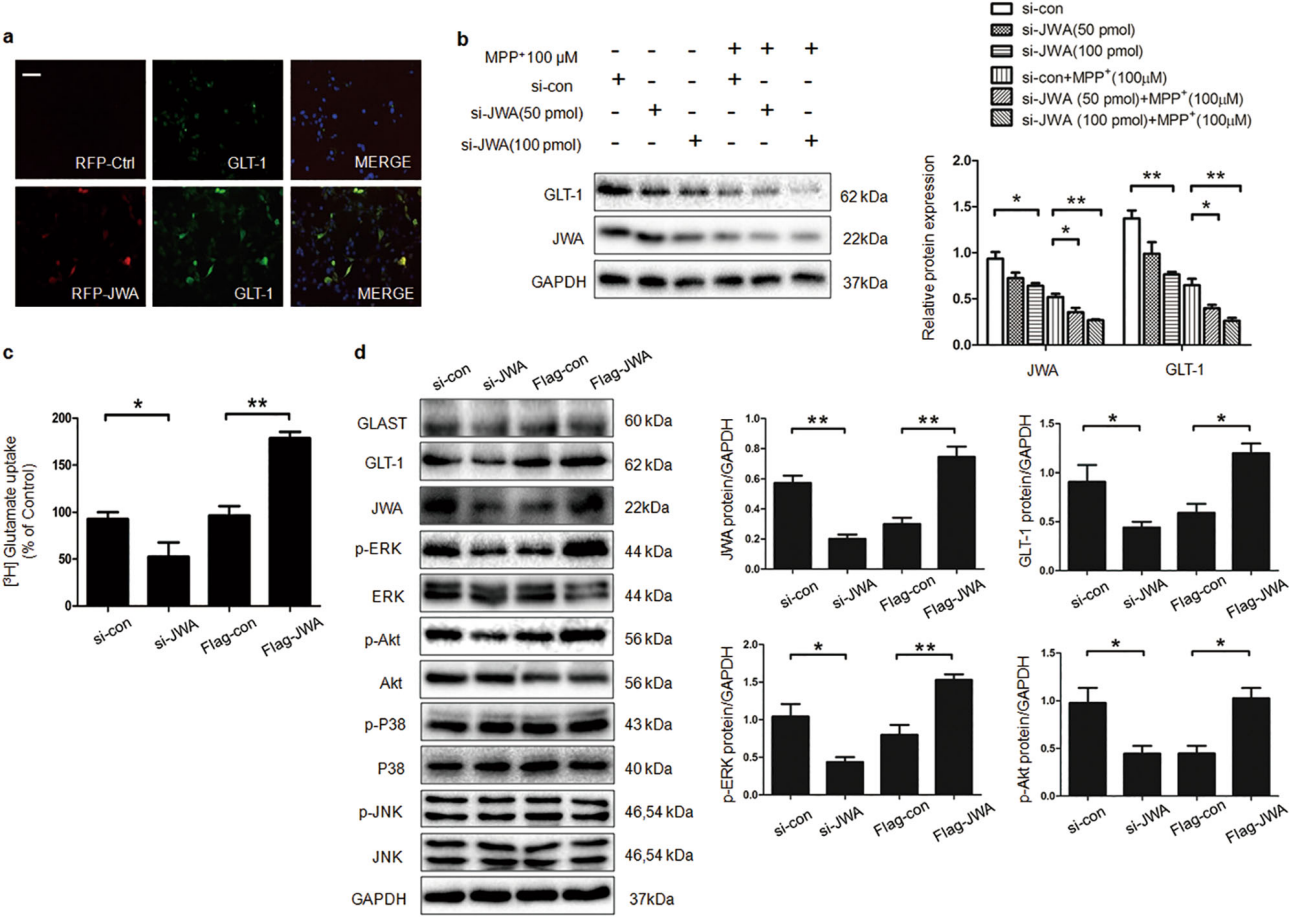
JWA regulates GLT-1 expression via ERK/MAPK and PI3K/Akt signaling pathways. **a** C8D1A cells were transfected with RFP-con or RFP-JWA. After 48 h, immunofluorescence for GLT-1 (green) was immunostained with anti-GLT-1 antibody. Scale bars = 200 μm. **b** C8D1A cells were transfected with si-con or si-JWA for various concentration (50 pmol or 100 pmol), followed by treatment with or without MPP^+^ 100 µM for 24 h. Expression levels of JWA and GLT-1 were detected by western blotting, GAPDH was used as a loading control to confirm that equal amounts of protein were loaded in each line (*n* = 3). **c** [^3^H]-Glutamate uptake assay was performed after C8D1A cells were transfected with si-JWA or Flag-JWA (*n* = 3). **d** C8D1A cells were transfected with si-JWA or Flag-JWA, followed by western blotting analysis to detect JWA, GLAST, GLT-1, phospho- and total-ERK, P38, JNK, and Akt proteins. The quantification for analysis of JWA, GLT-1, p-ERK, p-Akt protein (*n* = 3). Data are presented as mean ± SEM, one-way ANOVA, *P < 0.05, **P < 0.01

We used the mouse astrocyte cell line C8D1A to determine the function of JWA in GLT-1 expression. After 48-h transfection with RFP-con or RFP-JWA, immunofluorescent assay was used to test the effect of JWA overexpression on GLT-1. GLT-1 was significantly increased and colocalized with JWA (Fig. 5a). We next asked if 1-methyl-4-phenylpyridinium ion (MPP^+^) treatment affected regulation by JWA of GLT-1 expression. C8D1A cells were transfected with different levels of si-JWA (50 pmol or 100 pmol) for 24 h, and treated or untreated with 100 µM MPP^+^. The results showed that si-JWA greatly reduced GLT-1 expression with or without MPP^+^ administration (*P* < 0.05 or *P* < 0.01; Fig. 5b). Moreover, knocked down or overexpressed JWA significantly decreased or elevated glutamate uptake activity, respectively, compared with the vector control in C8D1A cells (*P* < 0.05 and *P* < 0.01, respectively; Fig. 5c).

To explore the regulation of JWA on GLT-1 in astrocytes, first, we performed immunoprecipitation assay to examine the direct interaction between JWA and GLT-1, EAAT3, which are expressed in astrocytes and neurons, respectively. In vivo and in vitro experiments showed that JWA only had direct interaction with EAAT3 but failed to directly interact with GLT-1 (Supplementary Figure 2). These results are consistent with previous studies^14^. Several studies show that various signaling pathways are involved in the expression of GLT-1, such as mitogen-activated protein kinase (MAPK), PI3K/Akt, and nuclear factor kappa-light-chain-enhancer of activated B cells (NF-κB)^20–23^. Here we investigated whether these signaling pathways mediate the effect of JWA on GLT-1 expression. The data revealed that there was a significant decrease of phosphorylated extracellular regulated protein kinases (p-ERK) and p-Akt in the JWA knock down group (*P* < 0.05). In contrast, JWA overexpression greatly increased p-ERK and p-Akt (*P* < 0.05 and *P* < 0.01, respectively). The levels of ERK and Akt were not affected, and p-P38 and p-JNK levels did not change in any group.

### Regulation by JWA of GLT-1 is mediated by p-CREB at the transcriptional level

**Fig. 6:**
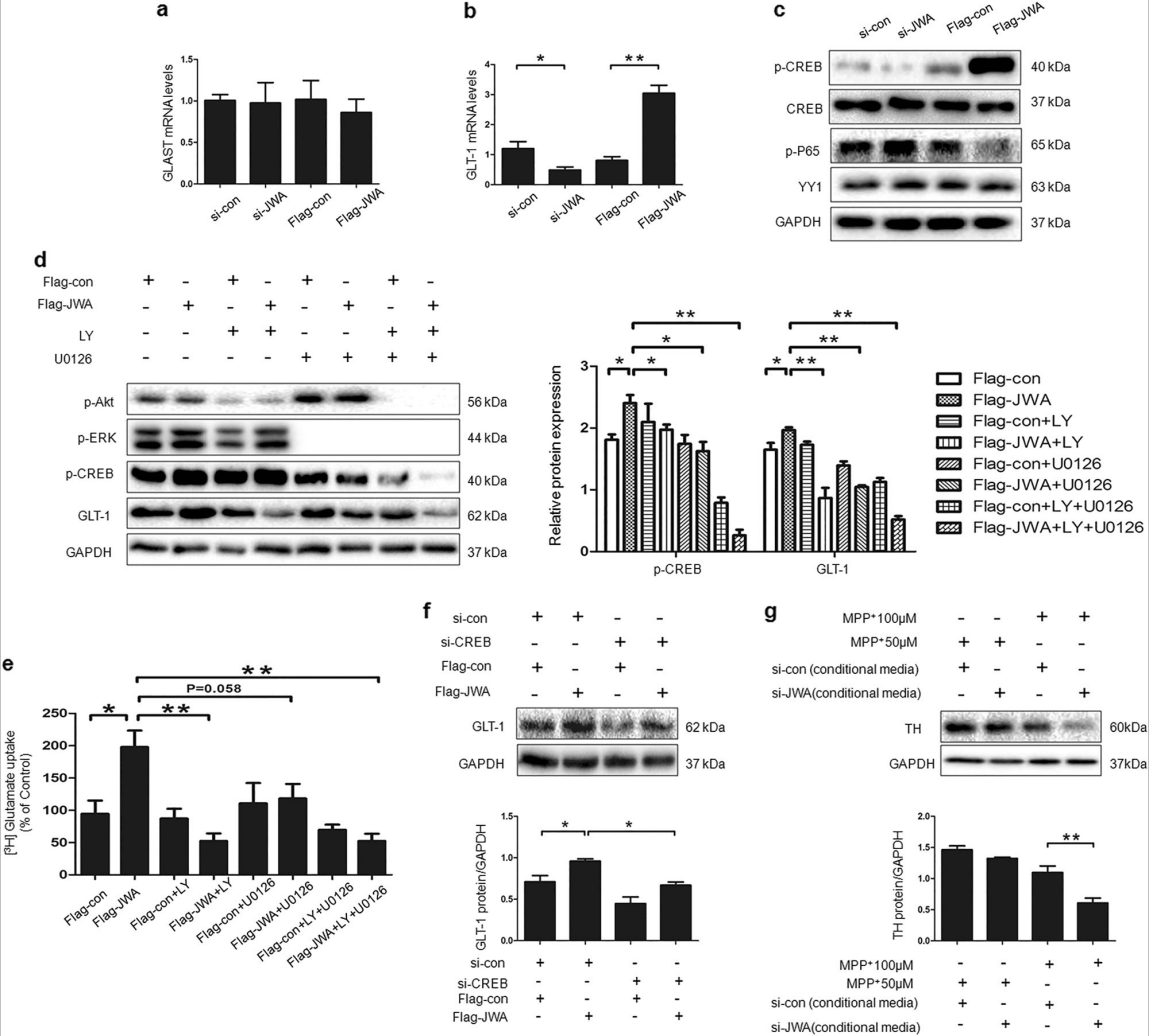
Transcription factor CREB is responsible for JWA-mediated GLT-1 expression. **a**, **b** C8D1A cells were transfected with si-JWA or Flag-JWA. After 48 h, the mRNA levels of GLAST and GLT-1 were measured by quantitative real-time PCR. GAPDH was used as a control to normalize the differences in the amount of total RNA in each sample (*n* = 3). **c** C8D1A cells were transfected with si-JWA or Flag-JWA, followed by western blotting analysis to detect transcription factor p-P65, YY1, phospho- and total-CREB proteins (*n* = 3). **d**, **e** C8D1A cells were incubated with 50 µM of U0126 and/or 20 µM of LY for 3 h after transfect with Flag-con or Flag-JWA for 48 h, followed by western blotting analysis to detect p-ERK, p-Akt, p-CREB and GLT-1 (*n* = 3). [^3^H]-Glutamate uptake assay was also performed (*n* = 3). **f** C8D1A cells were co-transfected with either Flag-con or Flag-JWA, together with the si-con or si-CREB. After 48 h, expression levels of GLT-1 were detected by western blotting, GAPDH was used as a loading control to confirm that equal amounts of protein were loaded in each line (*n* = 3). **g** SH-SY5Y cells were treated for 24 h with astrocyte CM from astrocytes transfected with si-con or from astrocytes transfected with si-JWA. After the treatment, SH-SY5Y cells were exposed to MPP^+^ (50 μM or 100 μM) for 24 h. The expression levels of TH were detected by western blotting, GAPDH was used as a loading control to confirm that equal amounts of protein were loaded in each line (*n* = 3). Data are presented as mean ± SEM, one-way ANOVA, *P < 0.05, **P < 0.01

To understand the mechanism of JWA regulation of GLT-1 expression, we measured the effect of JWA on GLT-1 mRNA levels in C8D1A. JWA knock down greatly reduced GLT-1 levels (*P* < 0.05), but GLT-1 levels increased after overexpression of JWA (*P* < 0.01). However, there was no effect on GLAST (Figs. 6a, b).

We determined transcription factors involved in JWA regulating GLT-1. As shown in Fig. 6c, knocked down or overexpressed JWA significantly downregulated or elevated, respectively, the levels of p-CREB, but p-P65 expression was negatively regulated by JWA. There was no effect on YY1. Accordingly, we applied specific inhibitors for MAPK/ERK and PI3K/Akt pathways to confirm the role of the signaling pathway on p-CREB and GLT-1 expression. The results showed that inhibition of the ERK (U0126) and Akt (LY294002) pathways abolished JWA-induced p-CREB and GLT-1 expression (*P* < 0.05 or *P* < 0.01; Fig. 6d). Consistent with the protein results, inhibitors significantly decreased glutamate uptake activity after 4-h incubation (*P* < 0.05 or *P* < 0.01; Fig. 6e). C8D1A cells were transfected with Flag-con or Flag-JWA for 24 h before transfection with CREB small interfering RNA (siRNA). GLT-1 was significantly suppressed by the CREB siRNA compared with the scrambled RNA control groups (Fig. 6f). These results indicate that CREB contributes to the JWA regulation of GLT-1 expression.

To explore whether the loss of JWA in astrocytes resulted in potential toxicity to DA neurons, SH-SY5Y cells were treated for 24 h with conditioned medium (CM) from astrocytes transfected with si-con or transfected with si-JWA. The cells were then exposed to MPP^+^ (50 μM or 100 μM) for 24 h. The CM from si-JWA astrocytes were less protective of neurons against MPP^+^ compared with CM from si-con astrocytes. MPP^+^ (100 μM) induced a significant 45% decrease of TH expression in si-JWA astrocytes CM treatment cells (Fig. 6e).

### Astrocytic JWA deficiency increases the expression of GFAP in various brain regions via the transcription factor signal transducer and activator of transcription 3 (STAT3).

**Fig. 7:**
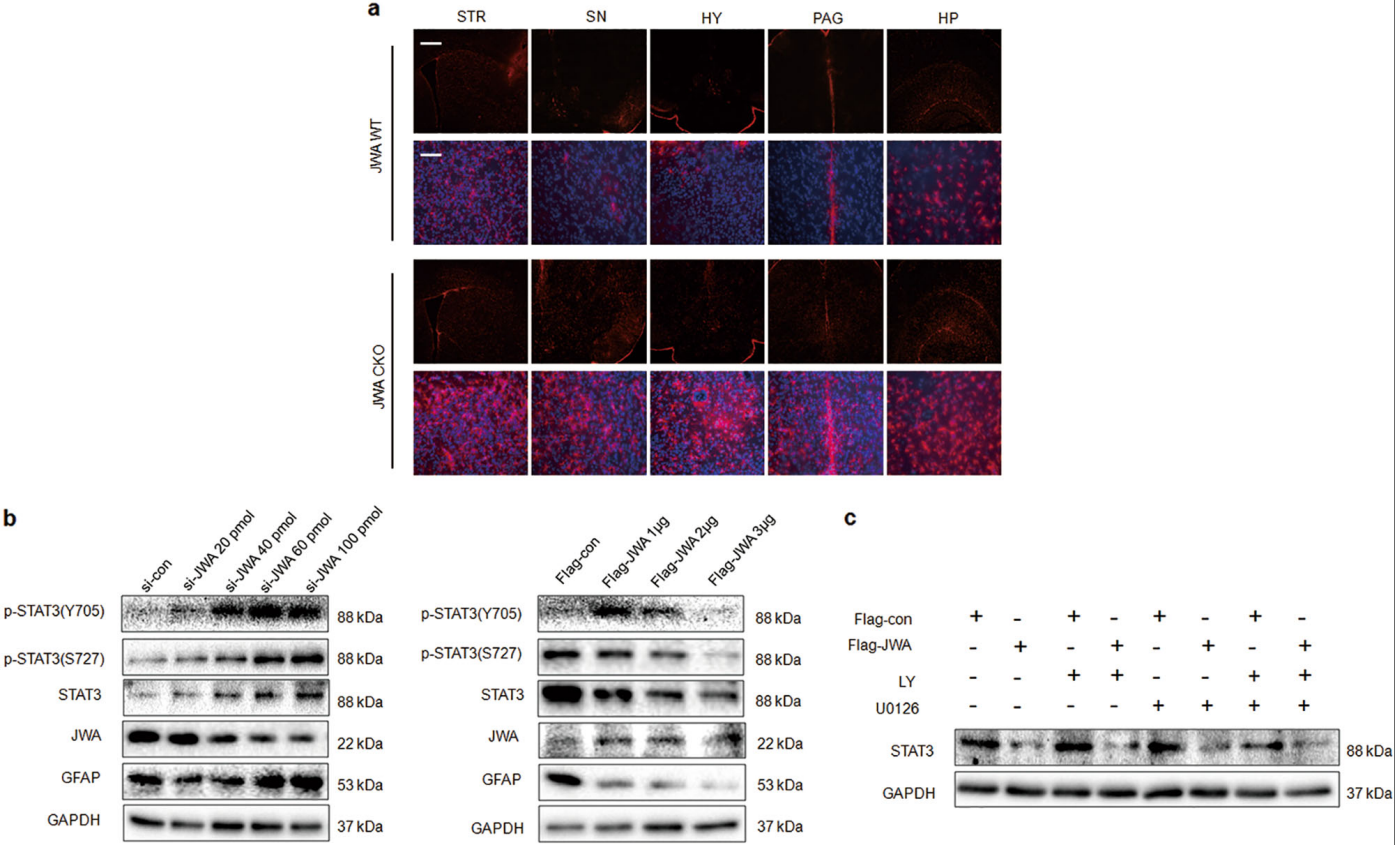
JWA regulates the expression of GFAP by transcription factor STAT3. **a** Immunofluorescence for GFAP (red) was immunostained with brain regions STR, SN, HY, PAG, and HP. Scale bars = 200 μm. **b** C8D1A cells were transfected with different levels of si-JWA (20, 40, 60, and 100 pmol) or Flag-JWA (1, 2, 3 µg) for 48 h, followed by western blotting analysis to detect p-STAT3(Y705), p-STAT3(S727), and STAT3 proteins. **c** C8D1A cells were incubated with 50 µM of U0126 and/or 20 µM of LY for 3 h after transfect with Flag-con or Flag-JWA for 48 h, followed by western blotting analysis to detect STAT3 (*n* = 3). GAPDH was used as a loading control to confirm that equal amounts of protein were loaded in each line

Abundant astrocyte reactivity with short and thick processes was observed in STR, SNc, hypothalamus (HY), periaqueductal gray (PAG), and hippocampus (HP) of JWA CKO mice. In contrast, astrocytes in these brain regions of JWA WT mice showed a resting phenotype (Fig. 7a). This indicated that astrocyte reactivity occurred not only in STR and SNc but also in various brain regions.

To identify if STAT3 is involved in JWA deficiency-induced astrocyte reactivity, we analyzed changes in p-STAT3 and STAT3 expression in C8D1A cells (Fig. 7b). The results showed that si-JWA greatly increased p-STAT3(Y705), p-STAT3(S727), and STAT3 levels, but overexpression of JWA had opposite effects and the effects were dose dependent. Additionally, we applied specific inhibitors for mitogen-activated protein kinase kinase (MEK) and PI3K to confirm the role of signaling pathways on STAT3 expression. The inhibitors of MEK and PI3K had no effects on the JWA-induced inhibition of STAT3 (Fig. 7c). These findings suggest that JWA may be involved in the phosphorylation of STAT3 through the negative regulation of STAT3 to induce astrocyte reactivity, which is independent on MEK and PI3K signaling pathways.

### Astrocytic JWA deficiency accelerates motor dysfunction, dopaminergic neuron degeneration, and impacts monoamine neurotransmitter levels via the nigrostriatal pathway in paraquat-treated mice

**Fig. 8:**
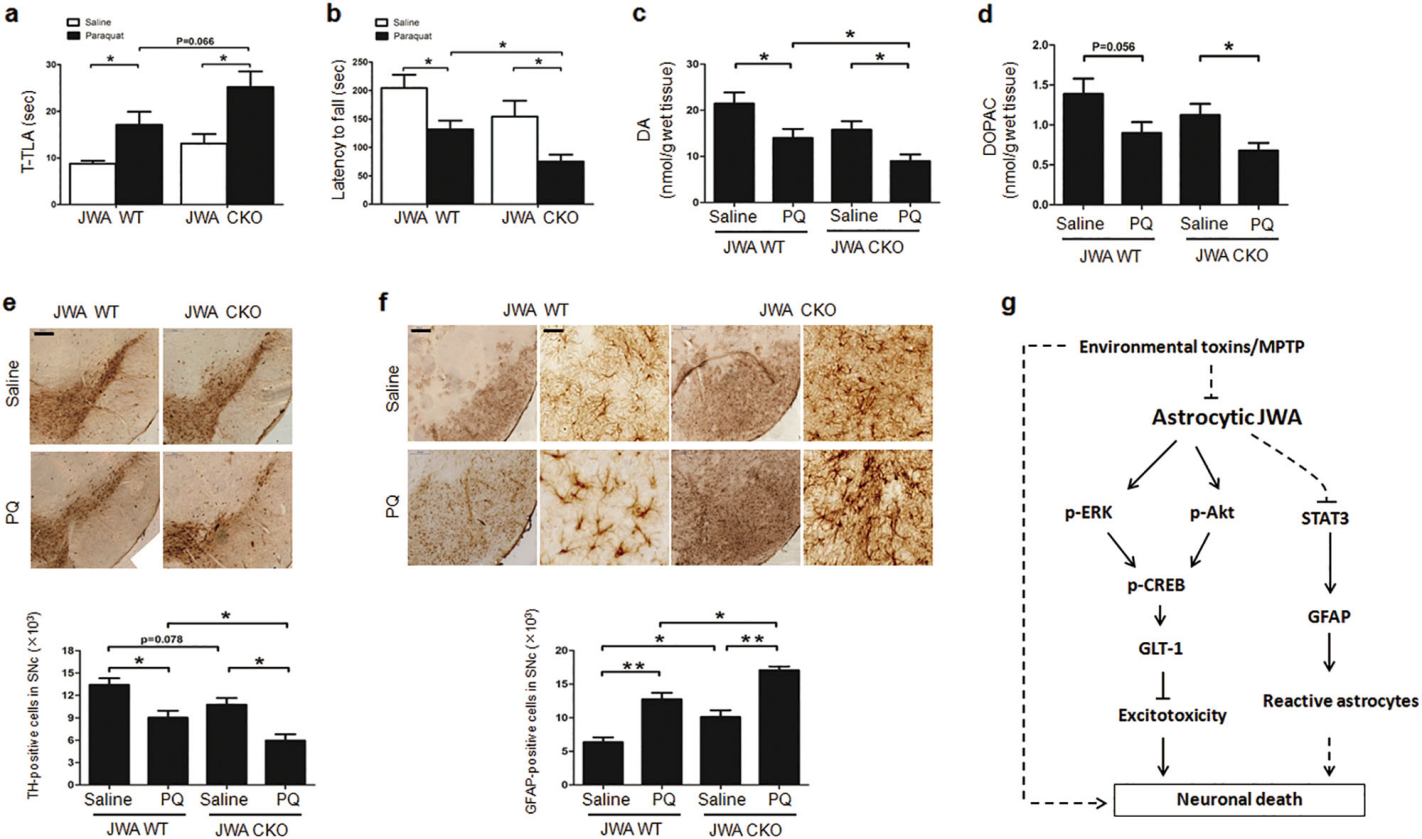
Exposure to PQ increases dopaminergic neuron degeneration in astrocytic JWA-deficient mice. **a**, **b** JWA WT and JWA CKO mice were trained 3 days on the rotarod until their motor performance was assessed. Behavioral assays of climbing time in pole test, the time taken for mice to descend a pole (T-TLA). The time spent on the rod was calculated (in seconds) (*n* = 15). **c**, **d** Levels of DA and DOPAC were analyzed by HPLC coupled to electrochemical detection in striatum tissues (*n* = 7–8). **e** Immunohistochemical staining and quantitative data for TH-positive neurons in the SNc (*n* = 4). Scale bar = 200 µm. **f** Immunohistochemical staining and quantitative data for GFAP-positive neurons in the SNc (*n* = 4). Scale bars = 200 μm (low magnification) and 50 μm (high magnification). Data are presented as mean ± SEM, one-way ANOVA, *P < 0.05, **P < 0.01. **g** A schematic diagram illustrates astrocytic JWA deficiency aggravates dopaminergic neuron death induced by environmental toxins

After paraquat (PQ) administration, JWA CKO mice exhibited a 1.38-fold increase of total time in the open-field test (*P* = 0.066). JWA CKO mice displayed a ~ 43 % reduction in the time spent on the rotarod compared with the PQ-treated JWA WT mice (*P < *0.05; Figs. 8a, b).

The results of monoamine neurotransmitters were similar to that of the MPTP/p model. PQ treatment decreased striatal DA by 36 % in JWA CKO mice compared with JWA WT mice (*P* < 0.05; Figs. 8c, d). Consistently, in comparison with the PQ-treated JWA WT mice, the number of TH-positive cells was reduced ~ 34 % in PQ-treated JWA CKO mice (*P* < 0.05; Fig. 8e). The GFAP-positive astrocytes were also examined in the SNc. In comparison with PQ-treated JWA WT mice, the number of TH-positive cells was increased by 1.34-fold in PQ-treated JWA CKO mice (*P* < 0.05; Fig. 8f). The effect of PQ on TH, GLT-1, and GFAP were more pronounced in JWA CKO mice. The mRNA level of GLT-1 was significantly decreased in PQ-treated JWA CKO mice compared with PQ-treated JWA WT mice (*P* < 0.05) (Supplementary Figure 3). Together with the data obtained with MPTP/p, these results emphasize the higher vulnerability of JWA CKO mice to conditions of neurotoxicity and demonstrate the importance of astrocytic JWA for the survival of dopaminergic neurons and normal DA production.

## Discussion

In the present study, deletion of JWA in astrocytes significantly exacerbated the loss of DA neurons in the PD mouse models. In addition, JWA deletion in astrocytes impaired glutamate uptake by reducing the expression of GLT-1. JWA induced GLT-1 expression through the ERK/Akt and CREB cascade. Astrocytic JWA deletion reactivated astrocytes by STAT3. These data indicated that JWA plays a neuroprotective role in astrocytes and may be a regulator of PD progression.

JWA expression level was reduced in the SNc of chronic MPTP/p-treated mice (Supplementary Figure 4). The reduced JWA expression may contribute to PD pathogenesis. Astrocytes provide nutrients for neuronal survival and perform a range of homeostatic maintenance functions^24,25^, and its dysfunction might lead to pathological changes in CNS^26^. In the present study, we showed that JWA CKO mice developed a PD-like phenotype with selective loss of dopaminergic neurons in the SNc and reduction of the levels of monoamine neurotransmitters in the STR.

Glutamate is considered as one of main excitatory neurotransmitters in the CNS. In humans, five subtypes of neurotransmitters were identified and named as EAAT1-5. These transporters are differentially localized in various brain regions. Subtypes GLAST and GLT-1 are found in the membranes of astrocytes. GLT-1 is responsible for over 90% of glutamate reuptake within the CNS^27^. The impairment of astrocytic glutamate transporters causes excitotoxicity and has been implicated in a variety CNS diseases such as PD, AD, stroke, epilepsy, and amyotrophic lateral sclerosis (ALS)^28^. GLT-1-deficient homozygous mice develop lethal spontaneous seizures^29^. The upregulation of GLT-1 attenuated epilepsy and contributes to ischemic tolerance^30,31^. Here, we demonstrated that JWA CKO mice showed lower GLT-1 expression compared with JWA WT mice in the SNc. After MPTP/p treatment, JWA CKO mice had more severe depletion of GLT-1 expression. However, the expression of GLAST did not change much. One possibility is due to GLAST is most prominent in the cerebellum, may maintain its levels in other brain regions. In contrast, GLT-1 is expressed to a lower level in the cerebellum^32^. Additionally, GLT-1 is the most sensitive to a wider scope of blockers than other subtypes^33^.

Multiple intracellular signaling pathways are involved in GLT-1 regulation. Previous studies found that GLT-1 expression is regulated by PI3K/Akt and ERK1/2 pathways^20^. Several compounds have been reported to mediate these signaling pathways activation in protection against cell death^22,23^. Akt and ERK1/2 are key regulators of GLT-1 expression. Consistently, we show that JWA exerts a positive regulating effect on GLT-1 levels. Moreover, JWA works as an activator of the ERK1/2 and Akt signaling pathways and glutamate uptake. This effect can be abolished by inhibitors of ERK1/2 and Akt, suggesting that the Akt and ERK1/2 signaling pathways are involved in JWA-mediated effects on GLT-1.

The GLT-1 promoter contains numerous transcription factor binding sites. Previous work suggested that regulation of GLT-1 expression was mediated by the transcription factor NF-κB, Yin Yang 1 (YY1), and CREB^23,34^. Here, we demonstrated that p-P65 expression is negatively regulated, but p-CREB is positively regulated by JWA. NF-κB activity is required for both tumor necrosis factor α (TNFα)-mediated inhibition and epidermal growth factor (EGF)-mediated induction of GLT-1 expression in glial cells^35^. The deficiency of JWA in astrocytes greatly enhanced NF-κB activation and triggered inflammatory response in mesencephalon tissue and U251 cells^19^. We hypothesize that NF-κB may be an important transcript factor that participates in JWA deficiency suppression of GLT-1 expression. However, JWA overexpression resulted in the high expression of GLT-1 and low expression of NF-κB. This result suggests that NF-κB is not involved in JWA regulated GLT-1 expression. YY1 is a ubiquitous transcription factor that plays an important role in the CNS, and can activate or repress gene transcription^36^. However, in the present study, YY1 was not affected by JWA in vitro. p-CREB modulates the expression of neuroprotective molecules^37^. It has been reported that CREB-mediated gene expression is impaired in the CNS of both PD mouse models and patients^38^. Additionally, CREB plays a critical role in Raloxifene and tamoxifen-induced GLT-1 expression^22,23^. Our findings showed that JWA exerts a positive regulating effect on p-CREB, and this effect can be abolished by the MEK and PI3K inhibitor. In addition, inhibition of CREB with siRNA led to abrogation of the effect of JWA on GLT-1 expression. Taken together, our present study suggests that GLT-1 was induced by JWA through the activation of ERK/Akt and CREB cascade.

Astrocyte reactivity is a sensitive marker of pathological conditions in the CNS^39^. A dominant feature of reactive astrocytes is cellular hypertrophy, with overexpression of the intermediate filament GFAP. The upregulation of GFAP is observed in neurotrauma or neurotoxicity^40^. Our study showed that the density of reactive astrocytes in JWA CKO mice was higher than that in JWA WT mice in various brain regions. This result suggests that GFAP may be negatively regulated by JWA. STAT3 translocates to astrocytic nuclei, binds to the GFAP promoter, and finally promotes the transcription of GFAP^41^. The STAT3 pathway has been reported to increase GFAP expression in models of acute injury and neurodegenerative disorder^42^. Consistent with these observations, GFAP and STAT3 are negatively regulated by JWA indicating that JWA deficiency reactivated astrocytes by upregulating STAT3. Our results also showed that the regulation of STAT3 by JWA was not through the ERK/PI3K signaling pathway. Previous studies have shown that JWA can promote the ubiquitination and degradation of transcription factor Sp1 and death receptor 4 (DR4) by the proteasome^43,44^. Based on this, we speculate that JWA may promote the degradation of STAT3 through the ubiquitin–proteasome degradation pathway, and further investigation is required to test this hypothesis.

Both mRNA and protein expression levels of GLT-1 are decreased in Alexander disease transgenic mice and with elevated GFAP expression, however, the behind mechanisms are unknown^45^. In this study, JWA provided a linkage between increased GFAP levels and downregulated GLT-1. Thus, JWA may act as a “modulator” in astrocytes, and should be investigated as a potential new therapeutic gene target for neurodegenerative disease.

Environmental chemicals such as pesticides are extensively used worldwide. Abundant epidemiological studies have identified a correlative link between sporadic PD and long-term pesticide exposure^46,47^. PQ is a widely used herbicide and is structurally similar to MPTP^48^. Exposure to PQ alone or together with other pesticides has been shown to cause dopaminergic neuronal loss in vitro and in vivo^49^. In this study, our data showed that the phenotypes of PQ exposure were similar with those of MPTP in JWA CKO mice.

In conclusion, we reported for the first time that astrocytic JWA exerts a neuroprotective role against MPTP and PQ mediated neurotoxicity in mice. The neuroprotective role of JWA in astrocytes was realized by ERK/Akt-CREB-GLT-1 signaling. Knocking down of JWA causes astrocyte reactivity by STAT3. Therefore, our data in the present study suggest the potential to develop astrocytic JWA as a therapeutic target for the treatment of PD.

## Materials and methods

### Antibodies and reagents

PQ dichloride (36541), MPTP (M0896), MPP^+^ (D048), probenecid (P8761) and poly-l-lysine (P7890) were purchased from Sigma-Aldrich (St. Louis, MO). l-[^3^H] Glutamic acid (NET490250UC) was purchased from PerkinElmer (Waltham, MA). LY294002 (LY) (S1105) and U0126 (S1102) were from Selleckchem (Houston, TX). GLAST (ab181036), GLT-1 (ab41621), p-CREB (ab32096), p-STAT3 (Y705) (ab76315), p-STAT3 (S727) (ab32143), and STAT3 (ab68153) antibodies were from Abcam (Cambridge, MA). ERK (4695), p-ERK (4370), Akt (4685), p-Akt (Ser 473) (4070), CREB (9197), P38 (8690), p-P38 (4511), JNK (9252), p-JNK (9255), and p-P65 (3031) antibodies were from Cell Signaling Technology (Beverly, MA). TH (T8700) antibody was purchased from Sigma-Aldrich (St. Louis, MO). GAPDH was from Beyotime Biotechnology (Shanghai). GFAP (MAB360) was from Merck Millipore (Darmstadt). The Dulbecco’s modified Eagle’s medium (DMEM) (12491-015), Opti-MEM (31985-070), Trizol reagent (10296010), Lipofectamine 2000 (11668027), Sodium pyruvate (11360070), and nonessential amino acid (11140050) were obtained from Life Technologies (Grand Island, NY). Secondary antibodies were all purchased from Beyotime Biotechnology (Shanghai).

### Animals and treatments

All studies were conducted according to the guidelines for Laboratory Animal Research of Nanjing Medical University, and were approved by the Institutional Animal Care and Use Committee of Nanjing Medical University. The astrocyte-specific JWA knockout mice were produced as described previously^19^. The genotypes of Loxp and GFAP-Cre^+^ are shown in Supplementary Figure 1. The genotype of JWA-CKO mice was determined by PCR. Post-mortem midbrain slices were stained by immunofluorescence to visualize JWA-CKO mice construction. Mice were maintained under conditions of a 12-h light/dark cycle at 23 ± 2 °C, humidity 55 ± 5% and were provided with food and water ad libitum in the animal research center of Nanjing Medical University.

Male 12-week-old JWA CKO mice (*n* = 30), and JWA WT mice (*n* = 30) were used at the beginning of all experiments. The chronic MPTP/p PD model was created as described previously^50^. Briefly, JWA-CKO and WT mice were injected subcutaneously with 25 mg/kg MPTP in saline and 1 h later intraperitoneally with 250 mg/kg probenecid in dimethyl sulfoxide (DMSO) for 10 times with intervals of 3.5 days and were sacrificed at 7 days after the last injection. The control mice were treated with saline and probenecid.

PQ was dissolved in saline and given to mice through intraperitoneal injection. Each mouse in the PQ-exposed groups was subjected to a total of 10 injections of PQ at a dose of 7 mg/kg at 2-day intervals. Control mice were injected with saline only.

### Behavioral examination

We performed behavioral examination 6 days after the final injection of neurotoxins or saline to examine if there were behavioral differences between JWA WT and JWA-CKO mice.

#### Open-field test

Locomotion activity of mice was detected in an activity monitor under bright illumination. Each mouse was placed in an open-top, square Plexiglas box (30 × 30 × 40 cm^3^) in a quiet room, and allowed to freely explore for 10 min. The traveled distance was measured for 10 min.

#### Rotarod test

A rotarod apparatus was used to measure motor performance and coordination. Mice were trained 2 consecutive days. On days 1 and 2, mice learned to stay on the rotarod at constant speed (15 r.p.m.) for 300 s. At day 3, motor coordination was assessed on the rotarod at five constant speeds (2-20 r.p.m.) for a maximum of 60 s at each speed. The latency time that each mouse was able to stay on the rod for each rotarod speed was recorded.

#### Pole test

To investigate bradykinesia, we conducted the pole test. Briefly, a rough-surfaced wooden pole (1 cm in diameter, 55 cm in height) was vertically placed on the floor of a cage. Mice were placed head-upward on the top of the pole. Each mouse was accustomed to the apparatus the day before testing and allowed to descend three times. The test trials measured the total time until the mouse reached the floor with its four paws time-total latency (T-TLA). If the mice fell, or slipped down, a default value of 120 s was used. The test trials were performed three times per animal and the average value of the three examinations was used for each animal.

### HPLC determination of striatal DA and metabolites

DA and metabolites in the STR were measured by HPLC. Briefly, under chloral hydrate anesthesia (400 mg/kg, i.p.), STR tissues from eight mice were sonicated in chilled 0.1 M perchloric acid containing dihydroxybenzylamine as an internal standard. Tissues were prepared for HPLC/electrochemical determination (ECD) measurement of DA, DOPAC, and HVA contents. The HPLC/ECD system (Thermo, Shandon, Pittsburgh, PA, USA) consisted of a pump, an autosampler, a solvent delivery system, and a Coulochem III detector equipped with a Model 5300 CouloChem III, a Model 5041 analytical cell, and a Model 5020 guard cell. The mobile phase consisted of 75 mM Na_2_HPO_4_, 1.7 mM octane sulfonic acid, 0.05 M citrate, 0.05 M ethylene diamine tetraacetic acid (EDTA), 10% methanol, and 1.0 mM-heptanesulfonic acid. Concentrations were normalized by wet tissue weight.

### Cell cultures

The mouse cerebellum astrocyte cell line C8D1A was purchased from the China Center for Type Culture Collection (Wuhan, China). The C8D1A cell line was grown in DMEM supplemented with 5 mM sodium pyruvate, 5 mM nonessential amino acid, Ciprofloxacin (20 mg/ml), and 10% fetal bovine serum. Cells were maintained at 37 °C in a saturated humidity atmosphere containing 95% air and 5% CO_2_.

### Isolation of primary astrocytes

Astrocytes were isolated from the midbrain of 1-day-old C57BL/6 mice as previously described^51^. Briefly, neonatal mice were killed by rapid decapitation, the midbrain was removed and separated from the meninges and basal ganglia, and the tissue was cut into small pieces with scissors. All the tissue was then collected, resuspended in 10 ml of DMEM containing 0.25% trypase at 37 °C, and terminated by the addition of DMEM supplemented with 10% fetal bovine serum. After centrifugation at 1000 × *g* for 5 min, the cell pellets were resuspended and plated in six-well plates. The cultures were maintained at 37 °C in a 95% air, 5% CO_2_ incubator. The next day, the medium was changed and then renewed twice a week. When confluent, the primary cells were split and plated for required experiments.

### Cell transfections and treatments

The details of Flag-JWA and the control plasmids were described in a previous study^52^. RFP-JWA plasmids were constructed by modifications of the Flag-JWA plasmid. The JWA (5′-CGAGCTATTTCCTTATCTC-3′) and CREB (5′- CTGCCACAAATCAGATTAA-3′) siRNAs and a nonspecific control siRNA were synthesized by Ribobio (Guangzhou, China). C8D1A cells or primary astrocytes were cultured at a confluency of 70–80% in six-well plates and transfected with siRNA and plasmid DNA in OptiMEM using Lipofectamine TM 2000 Transfection Reagent according to the manufacturer’s protocol.

The MEK inhibitor U0126 and the PI3K inhibitor LY294002 (LY) were dissolved in DMSO; 50 µM of U0126 and 20 µM of LY were used in the experiments. MPP^+^ was dissolved in phosphate-buffered saline (PBS). In experiments, C8D1A cells were incubated with U0126 and/or LY for 3 h after transfection with Flag-con or Flag-JWA for 48 h. For MPP^+^ treatment, C8D1A cells were treated with MPP^+^ at 100 µM for 24 h after transfection with si-con or si-JWA at a concentration of 50 pmol or 100 pmol for 24 h.

To prepare primary astrocyte CM, primary astrocytes were transfected with si-con or si-JWA for 24 h. CM were collected, centrifuged at 3000 × *g* for 5 min to remove cellular debris, and the supernatants were stored at −80 °C until use. Human dopaminergic neuroblastoma SH-SY5Y cells were treated with primary astrocyte CM for 24 h. After the treatment, SH-SY5Y cells were exposed to MPP^+^ (at 50 μM or 100 μM) for 24 h and then detected by immunoblot assay.

### RNA preparation and quantitative real-time PCR

Total RNA was extracted from midbrain tissues (*n* = 6) and cells using the Trizol reagent. Next, 1 μg of total RNA was transcribed using a high capacity complementary DNA reverse transcription kit (Takara Biomedical Technology). qPCR was carried out using an ABI Prism 7900HT Real-Time PCR System with the following sets of primers, forward: 5′-GCCAACAATATGCCCAAGCAG-3′ and reverse: 5′-GACACCAAACACAGTCAGTGA-3′ for GLT-1; forward: 5′-CCATCCAGGCCAACGAAACA-3′ and reverse: 5′-GCTTCATTGAGAGAGTCGAAGAA-3′ for GLAST; forward: 5′-GCCGGTGCTGAGTATGTC-3′ and reverse: 5′-CTTCTGGGTGGCAGTGAT-3′ for GAPDH; forward: 5′-GGAGGAGTCATTGTGGTGC-3′ and reverse: 5′-GAAGTCTCAGGGATGCGTG-30 for JWA.

The following thermal cycling conditions were used: denaturation, 94 °C for 5 min followed by 35 cycles of denaturation at 94 °C for 35 s, annealing at 56 °C (for JWA) or 59 °C (for GAPDH, GLT-1, and GLAST) for 30 s and extension at 72 °C for 35 s. The cycles were followed by a final extension step at 72 °C for 8 min, and melting curves from 70 to 90 °C were determined.

### Immnohistochemistry and immunofluorescence

Endogenous peroxidase activity was quenched by incubation in 3% hydrogen peroxide in methanol for 30 min. The sections were blocked for 1 h with blocking solution (0.3% Triton X-100 and 5% bovine serum albumin in PBS). These sections were incubated overnight with primary antibody against TH (1:2000) and GFAP (1:1000). After washing in PBS, the sections were incubated in horseradish peroxidase-labeled goat anti-mouse/rabbit IgG antibody (1:1000) for 1 h at room temperature. The peroxidase reaction was performed in PBS using 3,3-diaminobenzidine tetrahydrochloride (DAB). Specimens were observed and counted using MicroBrightField Stereo Investigator software (Micro Bright Field, Williston, VT, USA) to measure the TH-positive cells and GFAP-positive cells.

For immunofluorescence, sections and cells were incubated with primary antibody against GFAP (1:1000) or GLT-1 (1:1000) overnight at 4 °C, and then labeled with an anti-rabbit secondary antibody conjugated with Cy3 or FITC (1:1000) in the dark for 1 h at room temperature. Cell nuclei were counterstained with 40, 60-diamidino-2-phenylindole (DAPI). Images were acquired by fluorescent microscopy (IX 70, Olympus, Japan). The cell confocal images were acquired with a Zeiss LSM 700 confocal microscope system (Carl Zeiss Jena, Oberkochen, Germany).

### Tissue preparation and western blotting

Under chloral hydrate anesthesia (400 mg/kg, i.p.), mice midbrain tissues of each group were dissected on ice after sacrifice. Midbrain and cell protein lysates were prepared with a radio immunoprecipitation assay buffer (RIPA) containing protease inhibitor cocktail (Sigma-Aldrich). The protein concentration in the lysate was determined by bicinchoninic acid assay (BCA). In all, 40 µg of each protein sample were electrophoresed through a 10–15% sodium dodecyl sulfate-polyacrylamide gel and blotted to polyvinylidene difluoride membrane, respectively. The membranes were probed with the primary antibodies and secondary antibodies. The blots were detected by an enhanced chemiluminescence western blotting detection kit. Optical densities of bands were analyzed by using Image J. Protein levels were quantified by computer analysis as the ratio between each immunoreactive band and the levels of GAPDH.

### Immunoprecipitation assay

The cells or brain tissues were washed with PBS twice, then pre-cooling immunoprecipitation (IP) buffer was added at 4 °C for 30 min. Next, the cells or tissues were centrifuged at 12,000 × *g* for 15 min at 4 °C. The supernatant was immediately transferred to a new centrifugal tube, and then anti-JWA antibody, anti-Flag, anti-EAAT3, and anti-GLT-1 antibody and appropriate control IgG (mouse, rabbit IgG, corresponding to the host species of the primary antibody) were added in 500 µg total protein and cultured at 4 °C. After 1 h, cell lysate was mixed with 20 µl of resuspended volume of protein A/G Plus-Agarose (Santa Cruz, USA) at 4 °C overnight. Afterwards, precooled IP buffer was used to wash the cells four times at 1000 × *g* for 5 min at 4 °C. The immunoprecipitate was then collected by centrifugation and analyzed by sodium dodecyl sulfate–polyacrylamide gel electrophoresis.

### Measurement of glutamate uptake

Glutamate uptake experiments were performed according to the procedure as described previously^53^. Briefly, cells were treated in Opti-MEM for the indicated times and then washed twice with preheated PBS buffer. Uptake assays were initiated by addition of L-[^3^H] glutamic acid to 24-well plates containing cell culture (0.25 mCi/mL) and incubated for 15 min at 37 °C. The cells were then lysed immediately with ice-cold 0.2 M NaOH. After centrifugation at 10,000 × *g* for 15 min, the supernatant was abstracted and mixed with scintillation fluid. Radioactivity was determined by liquid scintillation counting. The uptake activity was calculated as nmol glutamate/mg protein/min after correcting for protein levels.

### Statistics

Statistical differences were determined by Student’s *t*-test for two-group comparison or one-way analysis of variance test followed by Tukey’s post hoc test for multiple comparisons. The accepted level of significance was *P* < 0.05. The data in the text and figures are presented as the mean ± SEM.

## Electronic supplementary material

supplementary material
