# Supplementary material for: Astrocytic JWA deletion exacerbates dopaminergic neurodegeneration by decreasing glutamate transporters in mice
Source: Cell Death Dis. 2018 Mar 2;9(3):352. doi: 10.1038/s41419-018-0381-8 (PMC5834463; doi:10.1038/s41419-018-0381-8)
Supplement: Supplementary file 1 — supplementary material [file 41419_2018_381_MOESM1_ESM.docx]

**
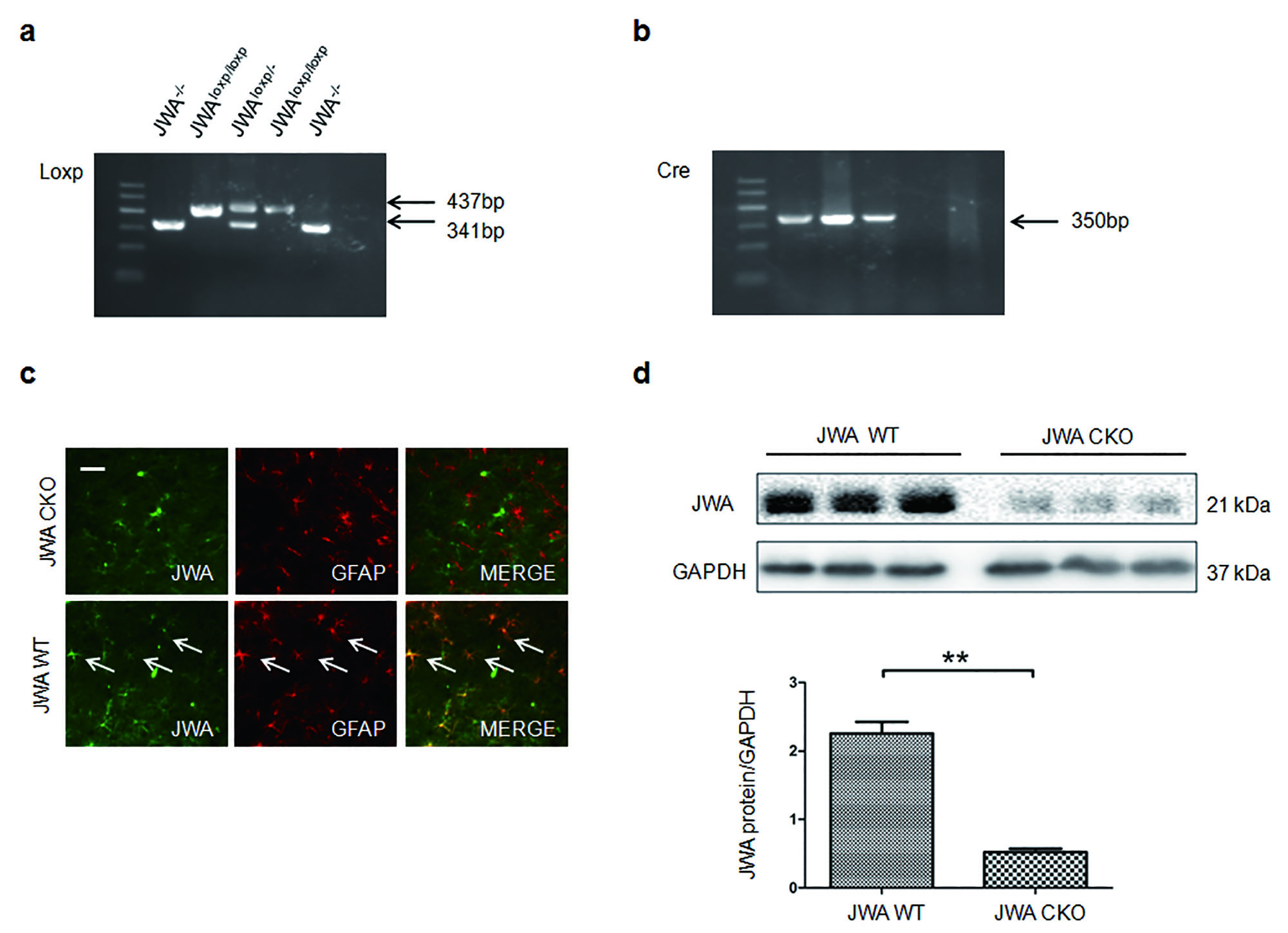
**

**Supplementary Figure 1** Genotype verification of astrocytic JWA deficient mice.

**a, b** Genotyping of JWA^-/-^, JWA^loxp/-^; GFAP-Cre^+^ and JWA^Loxp/Loxp^; GFAP-Cre^+^ mice by PCR. The JWA^Loxp/Loxp^; GFAP-Cre^+^ mice were identified with both 437 and 350 bp fragments; and the JWA^Loxp/Loxp^ mice were identified only with 437 bp band. **c** The immunofluorescence staining of JWA (green) and GFAP (red) expression in mice midbrain. Scale bars = 100 µm. **d** Immunoblots (upper) and quantification (lower) for analysis of JWA protein in JWA WT and JWA CKO mice (n = 3). Data are presented as mean ± SEM, one-way ANOVA, **p < 0.01.

**
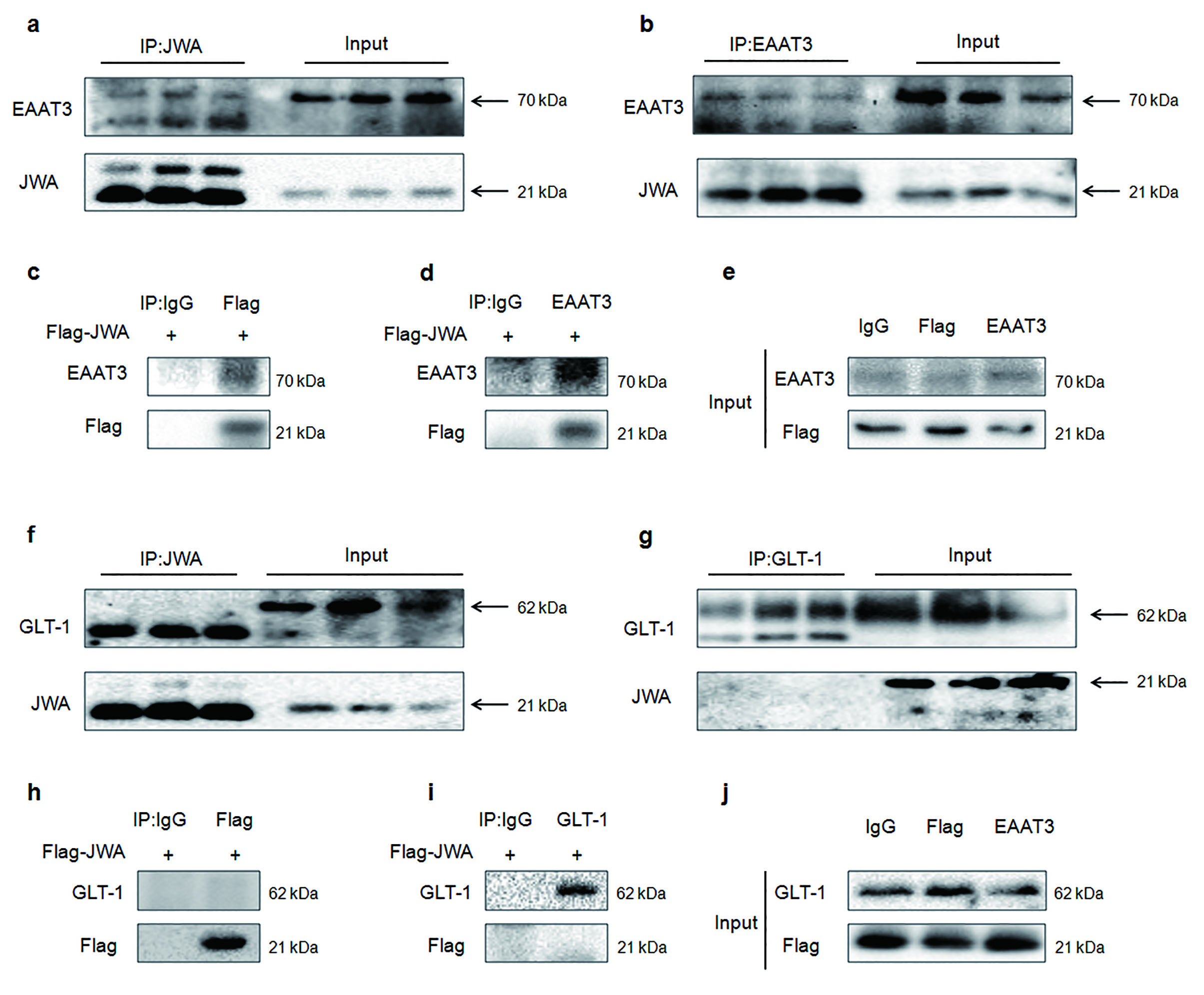
**

**Supplementary Figure 2** The direct interaction between JWA and GLT1, EAAT3. **a, b, f, g** Co-IP of JWA and GLT1, EAAT3 in brain tissues from the JWA WT groups with GLT1, EAAT3 or JWA antibodies followed by western blot (n = 3). **c-e** SH-SY5Y and **h-j** C8D1A cells transfected with Flag-JWA for 48 h, and the protein-protein interaction between EAAT3, GLT1 and JWA was assessed by Co-IP with EAAT3, GLT1or Flag antibodies followed by western blot (n = 3).

**
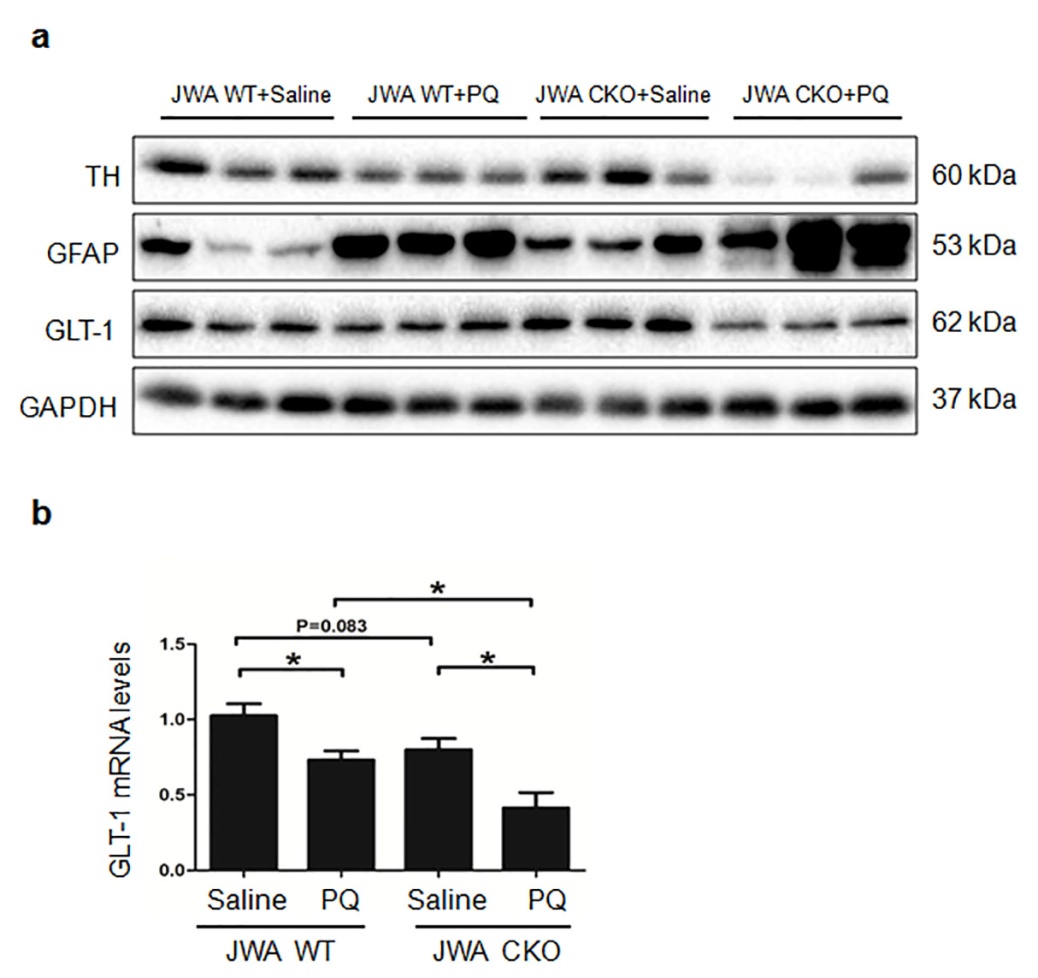
**

**Supplementary Figure 3** The expression of TH, GFAP and GLT-1 in SNc of paraquat-induced mouse model. **a** The expression of TH, GFAP and GLT-1 was detected by Western blotting using specific antibodies in midbrain extracts of paraquat-treated mice. GAPDH was used as a loading control to confirm that equal amounts of protein were loaded in each line (n = 6). **b** Quantitative real-time PCR analyses for GLT-1 is shown in the SNc. GAPDH was used as a control to normalize the differences in the amount of total RNA in each sample (n = 6). Data are presented as mean ± SEM, one-way ANOVA, *p < 0.05.

**
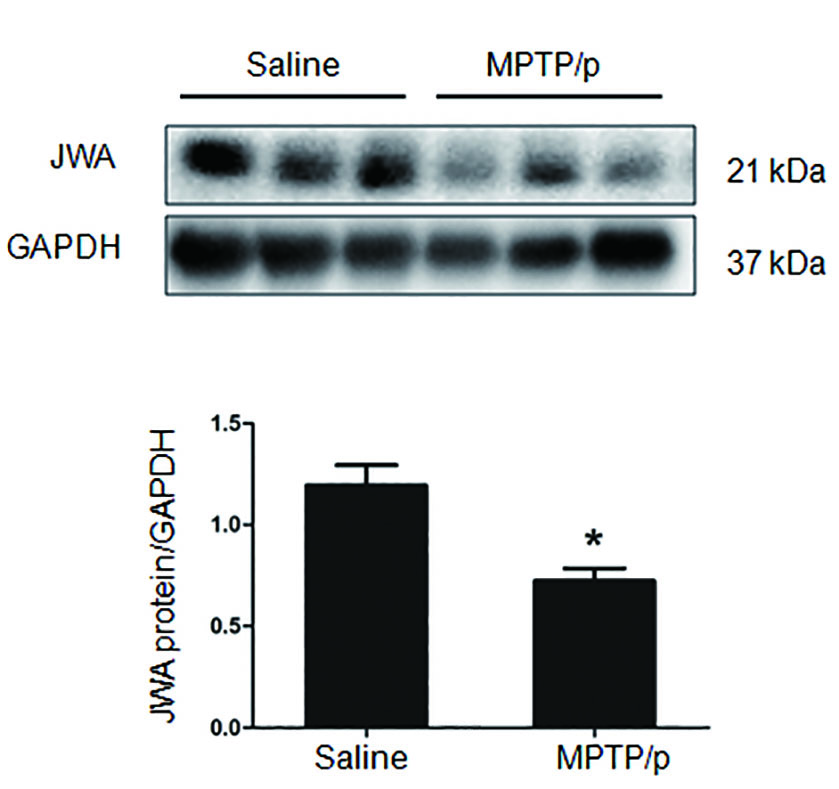
**

**Supplementary Figure 4** The expression of JWA in SNc of MPTP/p induced PD mouse model. Immunoblots (upper) and quantification (lower) for analysis of JWA protein in midbrain extracts of MPTP-treated mice (n = 3). Data are presented as mean ± SEM, one-way ANOVA, *p < 0.05.
